# Biomarkers for Alzheimer’s Disease: Context of Use, Qualification, and Roadmap for Clinical Implementation

**DOI:** 10.3390/medicina58070952

**Published:** 2022-07-19

**Authors:** Jeffrey Cummings, Jefferson Kinney

**Affiliations:** Pam Quirk Brain Health and Biomarker Laboratory, Chambers-Grundy Center for Transformative Neuroscience, Department of Brain Health, School of Integrated Health Sciences, University of Nevada Las Vegas, Las Vegas, NV 89154, USA; jefferson.kinney@unlv.edu

**Keywords:** Alzheimer’s disease, biomarkers, plasma, phospho-tau, amyloid, blood, neurofilament light, positron emission tomography, magnetic resonance imaging

## Abstract

*Background and Objectives*: The US Food and Drug Administration (FDA) defines a biomarker as a characteristic that is measured as an indicator of normal biological processes, pathogenic processes, or responses to an exposure or intervention. Biomarkers may be used in clinical care or as drug development tools (DDTs) in clinical trials. The goal of this review and perspective is to provide insight into the regulatory guidance for the use of biomarkers in clinical trials and clinical care. *Materials and Methods*: We reviewed FDA guidances relevant to biomarker use in clinical trials and their transition to use in clinical care. We identified instructive examples of these biomarkers in Alzheimer’s disease (AD) drug development and their application in clinical practice. *Results*: For use in clinical trials, biomarkers must have a defined context of use (COU) as a risk/susceptibility, diagnostic, monitoring, predictive, prognostic, pharmacodynamic, or safety biomarker. A four-stage process defines the pathway to establish the regulatory acceptance of the COU for a biomarker including submission of a letter of intent, description of the qualification plan, submission of a full qualification package, and acceptance through a qualification recommendation. Biomarkers used in clinical care may be companion biomarkers, in vitro diagnostic devices (IVDs), or laboratory developed tests (LDTs). A five-phase biomarker development process has been proposed to structure the biomarker development process. *Conclusions*: Biomarkers are increasingly important in drug development and clinical care. Adherence to regulatory guidance for biomarkers used in clinical trials and patient care is required to advance these important drug development and clinical tools.

## 1. Introduction

Alzheimer’s disease (AD) becomes increasingly common with aging, and the population of Americans aged 65 and older is projected to grow from 58 million in 2021 to 88 million by 2050 [1]. Five percent of people aged from 65 to 74, 13.1% of people aged from 75 to 84, and 33.2% of people aged 84 and older have AD dementia [1]. There are currently 6.5 million individuals with AD dementia in the United States, including 1.75 million aged from 65 to 74, 2.41 million aged from 75 to 84, and 2.31 million aged 85 and older [1]. In addition to those suffering from AD dementia, five million Americans manifest mild cognitive impairment (MCI) attributed to AD. AD is known to have a long pre-symptomatic phase that occurs before the onset of MCI. During this period, individuals have Alzheimer pathology changes in the brain that may eventually progress to a level of severity that induces cognitive, functional, and behavioral changes [2].

Progress in developing new treatments for AD has been limited. Five drugs were approved between 1993 and 2003, and aducanumab was approved in 2021. Symptomatic agents have modest clinical benefits in improving or delaying the emergence of cognitive, functional, and behavioral symptoms. They do not change the trajectory of the underlying biology of AD. Aducanumab was the first disease-modifying therapy (DMT) to be approved by the US Food and Drug Administration (FDA). Other monoclonal antibodies are poised to be considered for approval. The goal of treatment with DMTs is to preserve the patients at the highest level of function for the longest time. Initiating treatment during the pre-symptomatic phase of AD is intended to forestall the onset of symptoms; administering DMTs in the MCI phase of AD targets delaying the emergence of AD dementia; using DMTs in the treatment of AD dementia attempts to delay progression, preserve function, and maintain patient quality of life for as long as possible. Biomarkers play a key role in AD drug development, and progress in advancing more therapies that modify the course of AD depends on success of identifying an expanded repertoire of AD-relevant biomarkers and applying emerging biomarkers in clinical trials [3]. These measures of biological activity assist in the diagnosis, risk assessment, efficacy measurement, and safety evaluation of DMTs. Development of new biomarkers is subject to substantial FDA oversight through defined regulatory pathways. In this perspective, we review emerging biomarkers relevant to DMT drug development and AD treatment. We describe the regulatory pathways for advancing biomarkers for their use in clinical trials and their implementation in clinical care. We emphasize blood-based biomarkers because they have the fewest obstacles to use in the clinical care setting.

## 2. Materials and Methods

The goal of this review and perspective is to describe the FDA guidelines for the development of biomarkers as used in clinical trials as drug development tools (DDTs) and as used in clinical care. We identified the major relevant FDA guidances that present the definition of a biomarker, key requirements for biomarker qualification, and the development of companion biomarkers, in vitro diagnostic devices (IVDs), and laboratory developed tests (LDTs) for use in clinical care. Biomarkers for Research Use Only (RUO) are also regulated and subject to FDA oversight. We present examples of the application of these guidelines in the development of biomarkers for AD clinical trials and AD care.

## 3. Biomarker Definition and Classification

### 3.1. Biomarker Definition

The FDA defines a biomarker as a characteristic that is measured as an indicator of normal biological processes, pathogenic processes, or responses to an exposure or intervention [4,5].

### 3.2. Biomarker Classification

The FDA outlined the specific use of biomarkers in the Biomarkers, Endpoints and Other Tools (BEST) resource [5,6]. The BEST approach defines the following types of biomarkers: susceptibility or risk biomarkers, diagnostic biomarkers, monitoring biomarkers, pharmacodynamic biomarkers, predictive biomarkers, prognostic biomarkers, and safety biomarkers (Table 1).

### 3.3. Biomarkers in Alzheimer’s Disease

All the BEST categories of biomarkers are represented in the evolving repertoire of biomarkers available for use in characterizing AD biology and pursuing AD drug development. The context of use (COU; discussed below) for a biomarker must be defined prior to application in a trial. Some biomarkers may be used in several ways in a clinical trial. For example, amyloid positron emission tomography (PET) might be used as a diagnostic biomarker to confirm the diagnosis of AD, as a monitoring biomarker serially collected in trials of anti-amyloid monoclonal antibodies, and as a pharmacodynamic biomarker employed as an outcome in support of disease modification in a trial [7]. Similarly, in AD research, fluorodeoxyglucose (FDG) PET can be used to establish normal brain metabolism in an unaffected individual, demonstrate a pattern of reduction in the metabolism of an individual with AD, and measure a response to treatment that improves metabolism or reduces the rate of metabolic decline [8,9].

**Figure 1:**
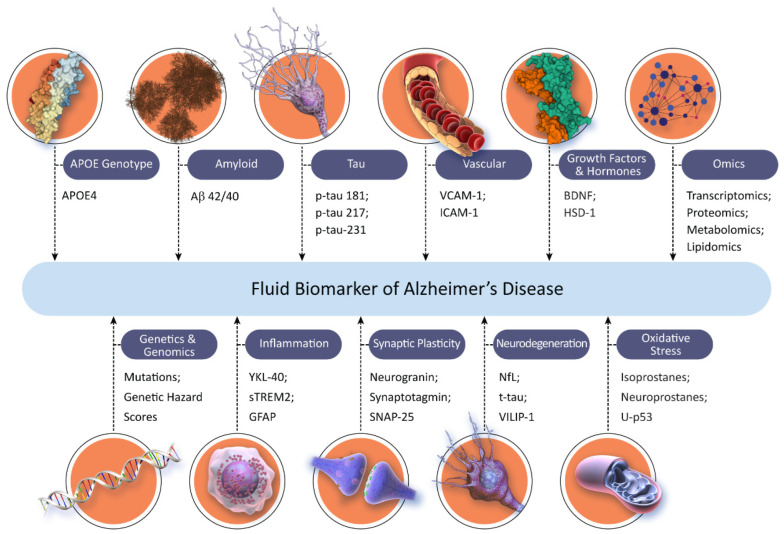
Landscape of fluid biomarkers for Alzheimer’s disease (© J Cummings; illustrator M de la Flor, PhD).

Figure 1 shows the current landscape of fluid biomarkers available for use in AD drug development (those shown are not an exhaustive list; new biomarkers are evolving rapidly; not all biomarkers shown are in the same state of development and some have more supportive data for their COU than others).

The existence of a biomarker does not imply that it will be an acceptable measure of drug effects in a clinical trial. Factors such as abundance in the blood or cerebrospinal fluid (CSF), dynamic range, change over time, intra-individual variability, population variability, and preanalytical factors may influence the potential to use a biomarker in a multisite trial. Similarly, a biomarker for treatment response might not be abnormal at baseline but could represent an important target engagement measure if changed by the intervention. For example, brain amyloid plaque levels measured by standardized uptake value ratios (SUVR) might be normal at baseline in a primary prevention trial and delaying the increase in SUVR could represent a trial outcome indicative of success in ameliorating amyloid accumulation.

Biomarkers are critically important in AD research and drug development because the brain cannot be directly interrogated, and tissue samples will rarely be available as they might be from tumor biopsies for use in oncology drug development. Biomarkers provide inferential evidence of pathological changes in the brain [10]. Fluid biomarkers are subject to metabolism and excretion influences like other metabolic products and drugs, and these affect the dynamics and measurement characteristics of peripheral biomarkers. Chronic kidney disease affects AD-related analyte excretion and is associated with higher levels of plasma biomarkers that could be mistakenly interpreted as indicative of AD [11]. Ethnic minority members often have higher levels of medical comorbidities, and these may affect biomarker levels and their interpretation [12]. Biomarkers collected from CSF are often more closely related to neuropathology than plasma biomarkers, and plasma–CSF discrepancies may reflect the peripheral processing of plasma biomarkers [13]. AD biomarkers have varying sensitivities for reflecting neuropathological findings, an observation that highlights the importance of collecting and reviewing more than one biomarker when using them as a basis for trial interpretation [14]. Biomarkers may be viewed as most clinically actionable when a positive or negative threshold can be defined. Such thresholds, however, have confidence intervals that condition their interpretation and a negative/positive read-out should be accepted with caution. An alternative is to define a high-confidence positive value, a high-confidence negative value, and an intermediate value where further assessment is warranted to interpret the biomarker or come to a clinical conclusion. An example of this strategy is the Amyloid Probability Score (APS) based on the plasma Aß 42/40 ratio, apolipoprotein E (APOE) genotype, and patient age that establishes thresholds for high, intermediate, and low likelihood of a positive amyloid PET scan [15].

### 3.4. Risk/Susceptibility Biomarkers

A risk biomarker indicates the potential for developing a disease or medical condition in an individual who does not currently have clinically apparent disease or medical abnormalities [4,5,6].

The most influential risk biomarkers for AD are mutations associated with autosomal-dominant AD (ADAD). Pathologic mutations of the amyloid precursor protein (APP) gene, presenilin 1 (PS1) gene, or presenilin 2 (PS2) gene are fully penetrant and, if present, lead to AD in the mutation carrier [16,17].

Carriers of the APOE ∈4 (APOE4) gene are at increased risk for the development of AD. Noncarriers of this gene have a lifetime risk of developing AD of approximately 15%, individuals who are heterozygous for the APOE4 gene have a lifetime risk for AD of approximately 30–40%, and persons homozygous for the APOE4 gene have a lifetime risk of 70–90% [18]. The risk conferred by the APOE4 gene appears to be attenuated in Black individuals [19]. Polygenic risk scores (containing single nucleotide polymorphisms (SNPs) identified as increasing the risk of AD in genome-wide association studies (GWAS)) explain up to 20% of additional risk beyond that associated with APOE4 [20].

Amyloid imaging can also be a risk marker. Not all individuals with an abnormal amyloid PET scan will progress to AD in their lifetime, and a having a positive amyloid PET can be regarded as a risk state for AD [21].

### 3.5. Diagnostic Biomarkers

Diagnostic biomarkers can be used to detect or confirm the presence of a disease or condition or to identify an individual with a subtype of a disease [4,5,6].

The diagnosis of AD requires the presence of biomarker-confirmed amyloid-beta protein (Aβ) in the brain [22]. This can be demonstrated by amyloid PET or CSF studies. PET studies show increased cortical plaque deposition, whereas CSF studies show a decrease in the monomeric form of Aβ. CSF levels of amyloid declines as the protein is progressively sequestered in plaques in the brain [23]. Recent studies of the clinical diagnosis of AD demonstrated that up to 40% of patients diagnosed with early AD (MCI and mild AD dementia) do not have pathologic levels of brain amyloid and do not meet biological criteria for AD [24]. Biomarker confirmation of the diagnosis of AD is critical to ensure that the target of anti-amyloid therapies is present for development programs targeting Aβ and to demonstrate that the diagnosis of AD is correct in programs advancing drugs targeting non-amyloid AD-specific disease processes.

Plasma biomarkers used to confirm the presence of AD are emerging. A reduced Aβ 42/40 ratio is consistent with the diagnosis of AD [25], and plasma tests for this ratio are commercially available (e.g., Precivity AD^TM^ and Quest AD-Detect^TM^). Plasma levels of phospho-tau (p-tau) 181 and p-tau 217 are elevated in patients with amyloid plaques and appear to be measures of plaque-related neuritic changes [26]. These tests might be used for the prescreening of individuals to identify those most likely to have a positive amyloid scan or anormal CSF amyloid studies. Biomarker panels of Aβ 42/40, p-tau, and measures of neurodegeneration such as neurofilament light (NfL) [27] combined with the APOE genotype may eventually be shown to be sufficiently accurate to diagnose AD without requiring CSF or PET confirmation.

Mutations of the APP, PS1, and PS2 genes cause ADAD (Loy, 2014). They are fully penetrant—in some cases (especially with PS2 mutations) the clinical syndrome may evolve late in life. The occurrence of an MCI or dementia syndrome in a person known to have an ADAD mutation and in whom other causes of cognitive impairment have been excluded (thyroid abnormalities, B12 deficiency, stroke, etc.) can be regarded as having a confirmed diagnosis of AD.

### 3.6. Monitoring Biomarkers

A biomarker that can be serially measured to assess the status of a disease or medical condition for evidence of exposure to a medical product or environmental agent or can be used to detect an effect of a medical product or biological agent is a monitoring biomarker [4,5,6]. Monitoring biomarkers are commonly used in clinical care and include serial measurements of blood pressure or cholesterol. Monitoring biomarkers can be important in ensuring the safe use of products through the serial assessment of liver functions, electrocardiograms, or other measures of organ function. Diagnostic markers, pharmacokinetic markers, and safety markers can all be used as monitoring biomarkers in specific circumstances. For example, amyloid PET imaging, p-tau measures, or magnetic resonance imaging (MRI) might be serially conducted to monitor efficacy or safety.

Monitoring biomarkers are increasingly used in AD drug development. For example, in trials of monoclonal antibodies (MABs), serial measurement with amyloid PET has shown increasing plaque reduction with increasing exposure to the MAB [28,29,30]. Serial measurements of p-tau 181, p-tau 217, and Aβ 42/40 have been used as monitoring biomarkers and demonstrate changes that occur in concert with plaque reduction induced by MABs. MRI is used as a monitoring biomarker and a safety biomarker to detect amyloid-related imaging abnormalities (ARIAs) in patients receiving MABs [31,32].

### 3.7. Pharmacodynamic/Response Biomarkers

Pharmacodynamic/response biomarkers change with exposure to a medical product or an environmental agent [4,5,6]. There are several applications of pharmacodynamic biomarkers, including the demonstration of target engagement in the early phases of drug development, the characterization of biological changes consistent with disease modification in later phases of drug development, use as a surrogate for clinical measures when fully validated, and—in special circumstances—as measures that are considered reasonably likely to predict clinical benefits to support the accelerated approval of an agent.

In AD trials, target engagement biomarkers demonstrate whether a pharmacologic agent has engaged the specific target of therapy. Pharmacodynamic biomarkers are also used as trial outcomes to determine whether an agent has a disease-modifying impact on AD. The lowering of amyloid plaque burden, as shown on amyloid PET, is regarded by the US FDA as a pharmacodynamic biomarker likely to predict a cognitive outcome. Plaque reduction was used in the accelerated approval of aducanumab [33]. Amyloid and tau biomarkers may function as either target engagement biomarkers, showing that the agent has directly or indirectly impacted Aβ- or tau-related processes, or as biomarkers providing evidence in support of disease modification. Their interpretation depends on the COU defined for the biomarker prior to the initiation of the trial.

Target engagement pharmacodynamic biomarkers are particularly important in Phase 2 of AD drug development. In this phase, a proof-of-concept (POC) for the hypothesis being tested is sought. Table 2 presents the Common Alzheimer’s Disease Research Ontology (CADRO) classification of drug targets in AD and related dementias (ADRD) created by the National Institute of Health/Alzheimer’s Association (NIH/AA) collaboration on the International Alzheimer’s and Related Dementia Research Portfolio (IADRP). The table presents biomarkers that link the CADRO class to the biological process on which they report.

Examples of target engagement pharmacodynamic biomarkers for amyloid biology include reduction in CSF Aβ by gamma-secretase inhibitors and beta-secretase inhibitors [34,35]. Gamma secretase inhibitors increase the Aβ 1–15/16 fragment, suggesting that this elevation may function as a target-engagement biomarker [36].

Peripheral measures of tau biology in AD include p-tau 181, p-tau 217, and p-tau 231 [37]. Total tau is measurable in plasma, and CSF and may reflect cell death and neurodegeneration [38]. Visinin-like protein-1 (VILIP-1) is an additional cell death reporter detectable in CSF [39]. Amyloid, tau, and cell death (neurodegeneration) biomarkers comprise the amyloid, tau, neurodegeneration (AT(N)) classification of biomarkers used to indicate disease state; they are discussed below (Table 3) [2].

Inflammation is a key element of AD, and plasma glial fibrillary acidic protein (GFAP), YKL 40, soluble triggering receptor expressed on myeloid cell 2 (sTREM2), and monocyte chemotactic protein-1 (MCP-1) measured in the CSF have promise as target engagement biomarkers for anti-inflammatory agents [40,41,42,43,44]. The PET imaging of activated microglia has focused on the development of ligands for the 18 kDa translocator protein (TSPO). This protein is not unique to glia, and more selective ligands are under development [45].

Target engagement biomarkers for synaptic function include CSF neurogranin; synaptotagmin; synaptosomal-associated protein, 25 kDa (SNAP-25); and growth-associated protein 43 (GAP-43) [42,46,47]. These may function as biomarkers in trials of agents affecting synaptic integrity. The PET imaging of synaptic vesicle glycoprotein 2A (SV2A), a presynaptic vesicle membrane present in virtually all synapses, provides a quantitative measure of synaptic density and its changes in the course of AD [48].

Vascular factors contribute to AD, and cell adhesion molecules detectable in plasma may reflect this vascular pathology. Soluble plasma vascular cell adhesion molecule-1 (VCAM-1) and intercellular adhesion molecule-1 (ICAM-1) are elevated in the plasma of patients with AD dementia [49]. The CSF/plasma albumin ratio can be used to assess the integrity of the blood–brain barrier (i.e., the neurovascular unit). This ratio has been found to be normal in most studies of AD but may be abnormal in other disorders from which AD must be differentiated [50].

Growth factors and hormones comprise a CADRO category. A meta-analysis of available studies showed that the level of brain-derived neurotrophic factor is decreased in the later stages of AD but not in early AD [51]. Structural MRI measures of hippocampal size and white matter measures of fractional and quantitative anisotropy have been proposed as measures of growth-factor effects in trials [52]. CSF 11-ß-hydroxysteroid dehydrogenase type 1 (HSD-1) has been used a target engagement biomarker to detect the effects of HSD-1 inhibitors [53].

Neurotransmitters and transmitter receptors represent a CADRO category. Nicotinic and muscarinic cholinergic receptors can be labeled with PET ligands [54,55]. The vesicular acetylcholine transporter (VAChT) can be labeled and visualized with PET [56]. PET ligands are available to assess the integrity of serotonin transporters in AD [57]. The dopamine transporter (DaT) can be labeled for use with PET or single-photon emission computed tomography (SPECT) imaging and can assist in distinguishing AD from dementia with Lewy bodies and Parkinson’s disease dementia; the latter two are characterized by dopamine transporter depletion [58].

Evolving biomarkers that have promise as target engagement reporters but are not yet fully established include plasma and CSF biomarkers of oxidative stress such as lipid peroxidation, isoprostanes, and neuroprostanes [59]. Plasma and CSF measures of u-P53 are considered measures of oxidation-induced protein changes in neuronal cells [60,61]. Lipid measures that may have a role as AD biomarkers or target engagement biomarkers include cholesterol (including 24S-hydroxycholesterol), oxysterols, fatty acids, and phospholipids [62]. Lipidomic assays may contribute vital information on a slate of lipid-related molecules but have thus far been relatively under-explored [63]. FDG has been used a measure of target engagement in studies of AD treatment with glucagon-like protein 1 (GLP-1) agonists [64]. Some specific microRNAs involved in the epigenetic regulation of protein synthesis have been shown to be decreased in blood from patients with AD, suggesting that specific microRNAs might function as target engagement biomarkers of epigenetic regulators [65].

Mechanistically nonspecific evidence of target engagement can be garnered from electroencephalography (EEG), evoked potentials, and functional MRI (fMRI) [66,67,68,69]. The restoration or slowing of deterioration of these measures suggest that circuit function has been beneficially affected compared to placebo, demonstrate that the drug has entered the brain, provide preliminary evidence in support of the POC of drug activity, and may show drug–placebo differences in smaller samples than those required to demonstrate clinical effects.

Many AD-related disease mechanisms and the associated impacts of the test agents have no pharmacodynamic target engagement biomarker. The development of these drugs is particularly challenging because long large trials may be necessary to determine the biological impact of the therapy and the absence of a more immediate target engagement biomarker means that no information is available to determine if such trials are warranted or to guide calculation of the necessary trial sample size. Increase in the number of accurate, reliable, valid, and scalable target engagement biomarkers is an unmet need for AD drug development.

Multiomic studies are an emerging area of biomarker development in AD. Genomic, proteomic, transcriptomic, metabolomic, and lipidomic profiles have been shown to be abnormal in AD [70,71,72,73]. These tools have promise because the measures reflect many levels of processing in the central nervous system and can be used to identify disturbed pathways and networks that may comprise targets for treatment. The identification of multiple affected networks may help guide combination therapy trials. Advanced bioinformatic skills are required to interrogate the large datasets, and consensus is evolving on best practices for these analyses.

The amyloid, tau, neurodegeneration (AT(N)) research framework [2] defines the most widely used suite of pharmacodynamic biomarkers supportive of disease modification (Table 3). Each of the members of the AT(N) framework can be measured with brain imaging, CSF biomarkers, and plasma or blood-based biomarkers. Amyloid levels can be measured by amyloid PET [74], CSF measures of Aβ 42/40, or plasma measures of Aβ 42/40 [15,25]. Tau protein in neurofibrillary tangles is measured with tau PET [75], and p-tau monomers are measured in CSF and plasma [76]. Evidence of neurodegeneration is provided by MRI atrophy or reductions in metabolism on FDG PET [77]. N-acetylaspartate (NAA) detectable with MR spectroscopy is largely sourced from neurons, and its decrease functions as a measure of nerve cell loss [78]. CSF and plasma measures consistent with neurodegeneration include total tau, NfL, and VILIP-1 [79,80,81]. The AT(N) framework is elastic and can expand to include additional biomarkers as more evidence of their accuracy and potential role in trials and care accrues [82].

The goal of disease modification is to prevent or slow neuronal loss that is the key to ameliorating cognitive and functional decline in AD and other neurodegenerative disorders [83]. Markers of neurodegeneration such as total tau, NfL, and VILIP-1 offer supportive information regarding whether neurodegeneration has been impacted and disease modification has occurred. Biomarkers related to neurodegeneration such as tau, amyloid, and inflammation can contribute to the weight of evidence in favor of disease modification.

Another application of pharmacodynamic biomarkers is their use in the accelerated approval of therapeutic compounds. This regulatory mechanism is used when clinical information from trials for treatment of a life-threatening illness is not complete and the changes in a biomarker demonstrated in the trial are considered reasonably likely to predict clinical benefits [84]. A post-approval trial to confirm clinical benefits can be required to support accelerated approval. A reduction in plaque amyloids demonstrated by amyloid PET—a pharmacodynamic response—was considered reasonably likely to predict clinical benefits from treatment with the anti-amyloid MAB aducanumab and was the basis for approval by the FDA [33].

### 3.8. Predictive Biomarkers

Predictive biomarkers are defined by the finding that the presence or change in a biomarker identifies an individual or group of individuals more likely to experience a favorable or unfavorable effect from exposure to a medical product or environmental agent [4,5,6]. Predictive biomarkers may be used in enrichment strategies in the design and conduct of clinical trials. Enrichment using predictive biomarkers is intended to make the therapeutic effect clearer by recruiting those individuals most likely to respond to treatment into the clinical trial. Predictive biomarkers must be distinguished from prognostic biomarkers. Prognostic biomarkers are associated with differential disease outcomes; predictive biomarkers discriminate those who will respond or not respond to therapy.

The APOE4 genotype is a predictive biomarker of ARIA in patients receiving treatment with an anti-amyloid MAB. In the clinical trials of aducanumab, for example, participants without an APOE4 gene had a 20% occurrence rate of ARIAs, heterozygotes for the gene had a 36% occurrence rate of ARIAs, and homozygotes had a 66% occurrence rate of ARIA [85].

Surrogate biomarkers are biomarkers whose performance has been fully confirmed and can serve as trial outcomes in place of clinical measures since their predictive value for clinical benefits is known. Surrogate status depends on demonstrating the relationship between the biomarker changes and clinical outcome across multiple trials and several mechanisms affecting the pathway and the biomarker [6]. There are no fully validated surrogate biomarkers for AD.

### 3.9. Prognostic Biomarkers

A prognostic biomarker is used to identify the likelihood of a clinical event, disease recurrence, or disease progression in patients with a disease or medical condition of interest [4,5,6]. Prognostic biomarkers are differentiated from susceptibility/risk biomarkers that identify the likelihood of the transition from a healthy state to disease. Prognostic biomarkers are distinguished from predictive biomarkers that identify factors associated with the effect of intervention or exposure. In clinical trials, prognostic biomarkers are routinely used as entry criteria to identify patients who are most likely to progress during the trial. Prognostic biomarkers influence the power to draw conclusions from a clinical trial by affecting the rate of progression or the number of events occurring in the placebo group.

Several biomarkers that provide prognostic information for AD have been identified. P-tau-181 and p-tau 217 elevations have been associated with progression from normal cognition to MCI and from MCI to AD dementia [76,86]. Neurofilament light and VILIP-1 are biomarkers of neurodegeneration and have been shown to have prognostic value for progression in patients with MCI or dementia due to AD [81,87]. GFAP, a marker of astrogliosis, predicts decline in those with subjective cognitive impairment [88]. Tau PET offers prognostic information and forecasts MCI and AD dementia progression [89,90]. Positive amyloid PET increases the likelihood of the development of MCI or dementia due to AD but is present in the brain for 15–20 years prior to the onset of cognitive symptoms. Many patients with brain amyloid to not show cognitive decline prior to death, and amyloid PET by itself does not provide strong prognostic information [21].

### 3.10. Safety Biomarkers

A safety biomarker is measured before or after an exposure to a medical intervention or environmental agent to indicate the likelihood, presence, or extent of a toxicity as an adverse event [4,5,6]. Commonly used safety biomarkers include measures of drug-induced changes in hepatic, renal, or cardiovascular function.

The MRI monitoring of ARIA is an important application of a safety biomarker in AD drug development and clinical care. Patients receiving anti-amyloid MABs may develop ARIA with edema (ARIA-E) or ARIA with hemorrhage (ARIA-H). This is particularly likely during the initial phases of treatment. MRIs are scheduled at routine intervals in the first months of therapy, and additional imaging is performed if symptoms suggestive of ARIA occur [31,32].

## 4. Biomarker Qualification

Biomarker qualification refers to the FDA process that establishes the evidentiary framework for use of a biomarker in a drug development program [4]. Experience with biomarkers in clinical trials frequently provides critically important data that inform the use of biomarkers in clinical care, and confidence in the biomarker is built through application in trials.

For a biomarker development effort to be successful, the biomarker must be clearly identified and characterize, and its method of measurement must be fully described. The evidence necessary for this process includes: (1) describing the drug development need, (2) defining the COU, (3) considering potential benefits if the biomarker is qualified for use, and (4) considering potential risks associated with the use of the proposed biomarker in a drug development program [4]. Risks arise from the consequences of false positives or false negatives regarding the identification of disorders important to a patient’s health.

**Figure 2:**
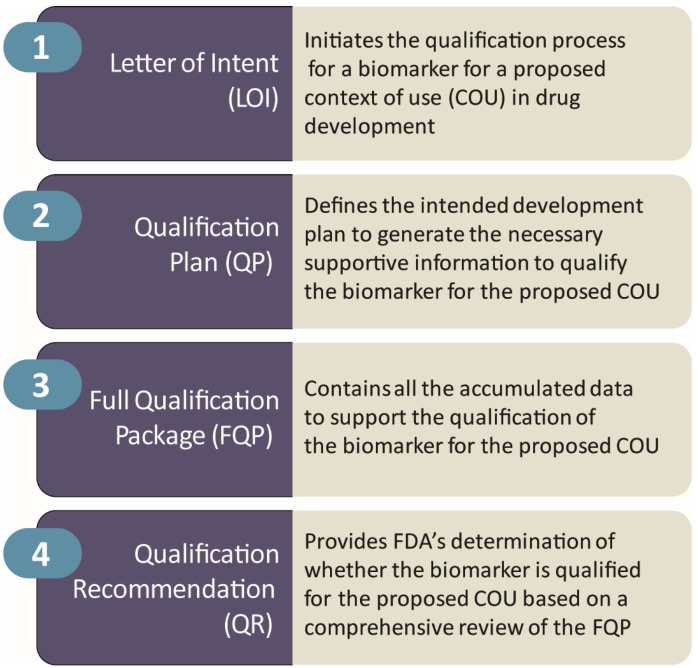
Context of use (COU) process required by the FDA for use of a biomarker as a drug development tool (DDT) in a clinical trial (© J Cummings; M de la Flor, PhD, illustrator).

A biomarker needs assessment describes why a biomarker is needed for drug development and how a biomarker might promote drug development in an area where there is an unmet medical need. The added value of the novel biomarker for the drug development process is described. The COU is a concise description of the biomarker’s specified use in drug development. The COU includes the identification of the type of biomarker (Table 1) and the proposed use of the biomarker in the drug development program. The COU process includes submitting a Letter of Intent (LOI) describing the intention to advance a biomarker COU, submitting a Qualification Plan (QP) that defines the intended development proposal to generate the necessary information to support the qualification of the biomarker, submitting a Full Qualification Package (FQP) that contains all the accumulated data to support the qualification of the biomarker, and obtaining a Qualification Recommendation (QR) that contains the FDA’s determination regarding whether the biomarker is qualified for the proposed COU [91]. Figure 2 presents the COU process required by the FDA. The potential benefits of a biomarker for use in a drug development plan depend on the biomarker’s proposed COU and the needs assessment. The potential risk of a biomarker depends on the consequences of incorrect decision making or harm to patients if the correlation between the biomarker and the outcome of interest are at variance.

The analytical validation of the biomarker must be presented as part of the proposed COU description [92]. The test’s reliability, validity, accuracy, sensitivity, specificity, precision, and reproducibility—as well as preanalytical factors such as collection, storage, and stability—must be determined before the COU can be approved. This information is included in the Full Qualification Package submitted for regulatory review.

## 5. Biomarkers for Use in Clinical Care

There are four clinical use pathways and one research pathway by which fluid biomarkers can be made available to clinicians for use in clinical care: as a companion diagnostic, as an in vitro diagnostic device (IVD), through the 510(k) pathway, as a Laboratory Developed Test (LDT), or as a test for Research Use Only (RUO). Table 4 lists and describes the five ways that biomarkers can be used in the clinical setting.

### 5.1. Companion Diagnostic

A companion diagnostic device is an IVD that provides information that is essential for the safe and effective use of a corresponding therapeutic product [84]. The use of an IVD companion diagnostic device with a therapeutic product is stipulated in the instructions for use in the labeling of both the diagnostic device and the corresponding therapeutic product. An IVD companion diagnostic device is considered essential for the safe and effective use of a corresponding therapeutic product to: identify patients who are most likely to benefit from the therapeutic product, identify patients likely to be at increased risk for serious adverse reactions as a result of treatment with the therapeutic product, monitor response to treatment with the therapeutic product for the purpose of adjusting treatment (e.g., schedule, dose, and discontinuation) to achieve improved safety or effectiveness, or identify patients in the population for whom the therapeutic product has been adequately studied and found safe and effective, i.e., there is insufficient information about the safety and effectiveness of the therapeutic product in any other population. This final category applies to patients with AD who are candidates for treatment with anti-amyloid MABs. Aducanumab has been studied only in patients with early AD, with amyloidosis confirmed by amyloid PET. The Appropriate Use Recommendations specify that the establishment of amyloid abnormalities through amyloid PET or CSF amyloid measures is required for the use of aducanumab since it is only in this population that the efficacy and safety of this agent have been studied [31,32]. Other MABs may be administered to restricted populations (early AD with positive amyloid studies) and may have similar requirements for safe and effective use.

### 5.2. In Vitro Diagnostic Devices (IVDs)

In vitro diagnostic devices (IVDs) include tools used to diagnose conditions and guide treatment decisions but are not required for the approved use of a specific product [93]. Unlike LDTs (discussed below), their measurement is not limited to a single laboratory. The test originators typically develop measurement kits that can be purchased and used in many laboratories. The terminology of “complementary diagnostic” may be used to describe a test that identifies a biomarker-defined subset of patients that respond particularly well to a drug and aid risk/benefit assessments for individual patients but are not prerequisites for receiving the drug. Complementary diagnostics are IVDs and are subject to the same regulatory requirements as other IVDs [94,95].

The FDA regulation of IVDs is risk-based: Class I tests pose relatively little risk to patients and the public health if they are inaccurate (such as a cholesterol test), Class II tests pose moderate risk if they are inaccurate, and Class III tests pose the greatest potential risk if they are inaccurate (an incorrect therapy could be chosen or a correct therapy could not be administered with severe health consequences) [93,96]. The three categories correspond to increasing levels of regulatory scrutiny.

Premarket approval (PMA) is required for some Class II tests and most Class III tests. PMA requires a demonstration of safety and effectiveness, including both analytical validity and clinical validity before the test is marketed. Analytical validity refers to how a test performs in detecting or measuring the presence of the analyte of interest. Analytically valid tests are precise, accurate, and reliable [92,93]. Clinical validity refers to how accurately a test predicts the presence of or risk for the condition of interest. The demonstration of clinical validity requires data from human testing and might include data generated in clinical trials. The FDA defines valid data in support of an IVD as evidence from well-controlled investigations, partially controlled studies, studies and trials without matched controls, well-documented case histories conducted by qualified experts, and reports of significant human experience with a marketed device from which it can fairly and responsibly be concluded by qualified experts that there is reasonable assurance of the safety and effectiveness of the device under its COU [97]. Laboratories performing tests on human specimens such as blood tests are subject to regulation under the Clinical Laboratory Improvement Amendments of 1988 (CLIA). This regulation governs the accreditation, inspection, and certification of clinical laboratories.

The Lumipulse-G measure of CSF Aβ 42/40 is an AD-related IVD approved for use in the US [98].

### 5.3. 510(k) Pathway

The 510(k) pathway is a variant of the IVD approval pathway. It is used if a test is substantially equivalent to a product already on the market. The sponsor provides evidence that the device has safety and efficacy characteristics at least equivalent to the existing approved IVD. Approval can be granted through a premarket notification process (510(k) pathway) [99].

### 5.4. Laboratory Developed Test (LDT)

Laboratory developed tests (LDTs) are biomarkers that are measured in a single laboratory and are available only from the identified resource [100]. Laboratories providing LDTs are CLIA-certified. LDTs are typically less rigorously scrutinized by the FDA than IVDs. Plasma Aβ 42/40 measures (Precivity AD^TM^ and Quest AD-Detect^TM^) are LDTs (available through C2N and Quest, respectively). If kits are created so an analyte can be assessed in other laboratories, an LDT could be re-classified as an IVD when sufficient data are available to satisfy FDA requirements.

### 5.5. Research Use Only (RUO) Test

Research Use Only (RUO) tests can be made available to clinicians and researchers to allow additional information regarding a biomarker’s performance or feasibility of use to be gathered. An RUO biomarker must be labeled as “not to be used for diagnosis” [101]. RUO biomarkers may be advanced to LDTs or IVDs with data development.

## 6. Five-Phase Roadmap for Biomarker Development

**Figure 3:**
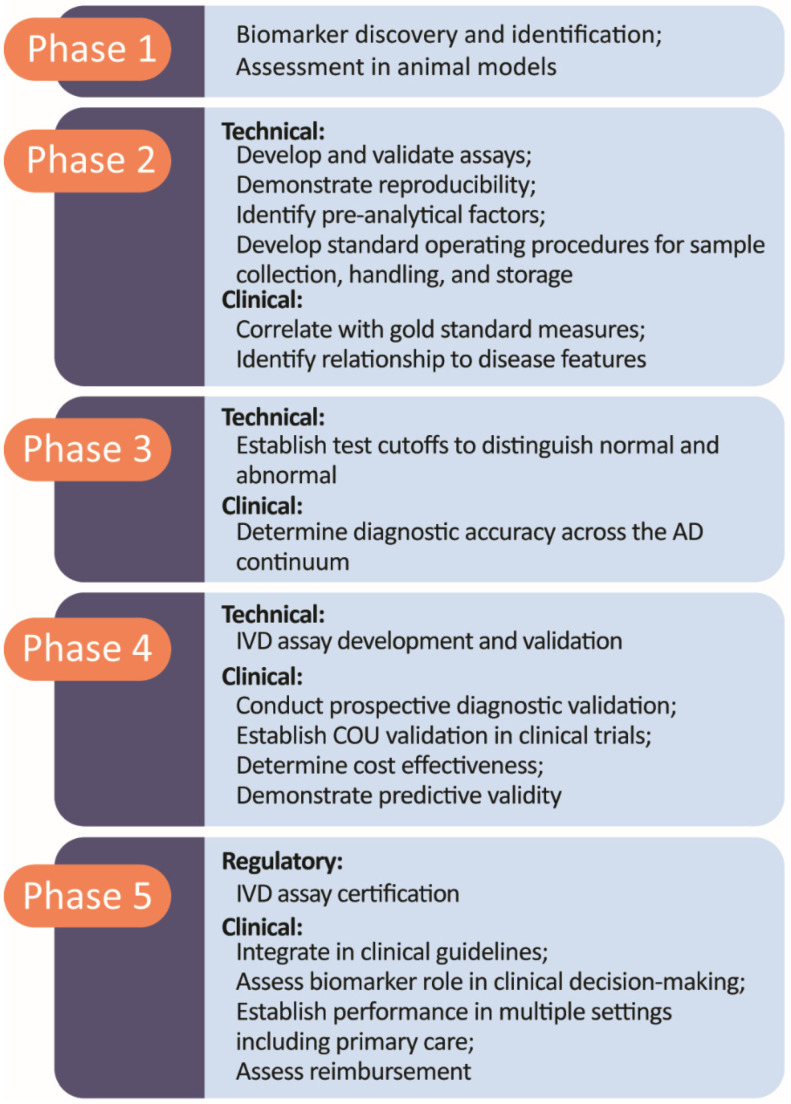
Five-phase process of biomarker development (© J Cummings, M de La Flor, PhD, Illustrator).

A European work group proposed a five-phase approach to IVD and diagnostic imaging data generation that begins with non-clinical exploratory studies (Phase 1), progresses to clinical assay development and validation (Phase 2), then advances to retrospective and longitudinal studies (Phase 3), moves to prospective studies and real world evidence (Phase 4), and concludes with implementation and studies of impact on clinical outcomes and cost-effectiveness, as well as the assessment of reimbursement (Phase 5) [102,103,104]. This pathway is based on the analysis of requirements for a biomarker to achieve routine clinical use and is not a regulatory requirement; it encompasses processes before and after regulatory review. Figure 3 shows the five phases of biomarker development. Phase 1 is the biomarker discovery phase based on the identification of biological processes that may have fluid or imaging markers. Phase 2 includes analytic validation and the preliminary analysis of accuracy in case control studies. Phases 2 and 3 provide evidence of clinical validity, and Phases 4 and 5 address clinical utility. Establishing a COU for a biomarker in trials typically occurs in Phase 4 after clinical validity has been demonstrated in Phases 2 and 3. Phases 4 and 5 provide the basis for widespread clinical use and reimbursement. Most AD biomarkers are in Phase 2 and 3, and some have established a COU for use in clinical trials. Few AD-related biomarkers have advanced to Phases 4 or 5 [103,105,106,107,108,109,110].

## 7. Biomarker Collaborations and Cohorts

An important challenge to biomarker development is accessing a sufficient number of well-characterized patients in whom the biomarker can be assessed and qualified. The Alzheimer’s Disease Neuroimaging (ADNI), Australian Imaging, Biomarker and Lifestyle Flagship Study of Ageing (AIBL), Amsterdam Dementia Cohort, and the BioFinder study host cohorts of well-studied patients that allow for the assessment of biomarkers [111,112,113,114]. Following the study of biomarkers in research centers, biomarkers require assessment in community-based practices to determine their robustness and utility in real world settings.

## 8. Conclusions

AD is a complex disease with many abnormal biological processes including amyloid accumulation, neurofibrillary tangle formation, neurodegeneration, inflammatory responses, and many other cell and network disturbances. These processes contribute to disease progression, and many of them may be targets for AD interventions. The clinical identification of these processes and the development of drugs to ameliorate them depends on biomarkers. Biomarkers for some processes have been developed, but many cell and network changes have no corresponding biological measure. The development of biomarkers for use in clinical trials and of IVDs and LDTs for use in clinical care is a critical part of the next step in the AD research agenda. Biomarker development requires rigorous data generation and regulatory review. Adherence to regulatory guidance for both biomarker development and introduction into the clinical setting is key to informative clinical trials and to successfully integrating biomarker use into clinical care settings.

## Figures and Tables

**Table 1:** FDA BEST classification of biomarkers use in drug development [5].

Biomarker	Measurement
Risk/susceptibility	Indicates the potential for developing a disease or medical condition in an individual who does not currently have a clinically apparent disease or medical condition
Diagnosis	Detects or confirms the presence of a disease or condition or identifies an individual with a subtype of the disease
Monitoring	Measured serially to assess the status of a disease or medical condition for evidence of exposure to a medical product or environmental agent or to detect an effect of a medical product or biological agent
Pharmacodynamic/response	Changes in response to exposure to a medical product or an environmental agent
Predictive	The presence or change in the biomarker predicts an individual or group of individuals more likely to experience a favorable or unfavorable effect from the exposure to a medical product or environmental agent
Prognostic	Identifies the likelihood of a clinical event, disease recurrence, or disease progression in patients with a disease or medical condition
Safety	Measured before or after an exposure to a medical intervention or environmental agent to indicate the likelihood, presence, or extent of a toxicity as an adverse event

**Table 2:** Target engagement biomarkers (CADRO—Common Alzheimer’s Disease Research Ontology); target engagement biomarkers are typically proximal in the cascade of events leading to cell death and dementia in AD. Biomarkers used to demonstrate disease modification using the amyloid, tau, neurodegeneration (A,T(N)) approach are listed in Table 3. Both well-established biomarkers and emerging, partially validated biomarkers are included in the table (the table is not an exhaustive list of all emerging biomarkers).

CADRO Category	Fluid Biomarkers	Imaging, Digital, and Device-Based Biomarkers
Amyloid beta	Inhibition of production of CSF Aβ by beta and gamma secretase inhibitors; increase in Aβ 1–15/16 by gamma secretase inhibitors	Amyloid PET
Tau	CSF and plasma p-tau 181, p-tau 217, and p-tau 231	Tau PET
APOE, lipids, lipoprotein receptors	Lipid peroxidation, isoprostanes, and lipidomics	None identified
Neurotransmitter receptors	None identified	Nicotinic cholinergic receptor PET, muscarinic receptor PET, dopamine transporter SPECT and PET, acetylcholine (VCHAT) and serotonin vesicular transporter PET
Neurogenesis	None identified	MRI measures of hippocampus; fractional and quantitative anisotropy
Inflammation	CSF and plasma GFAP, CSF YKL40, sTREM2, and MCP-1	TSPO PET and evolving ligands
Oxidative stress	Lipid peroxidation, isoprostanes, neuroprostanes, and u-P53	None identified
Proteostasis/proteinopathies	CSF Aβ and proteomics	None identified
Metabolism and bioenergetics	Metabolomics	FDG PET
Vasculature	Plasma VCAM-1 and ICAM-1; CSF/plasma albumin ratio to assess blood–brain barrier	MRI
Growth factors and hormones	Brain-derived neurotrophic factor (BDNF), HSD-1, and trial-specific hormones	MRI measures of hippocampal volume
Synaptic plasticity/neuroprotection	Neurogranin, synaptotagmin, and SNAP-25	SV2A PET
Cell death	Total tau, neurofilament light, VILIP-1, and GAP-43	Structural MRI (including hippocampal volume), FDG PET, and MR spectroscopy (NAA)
Gut-brain axis	Changes in blood amino acids and inflammatory cells	Changes in the microbe composition of the microbiome
Circadian rhythm	None identified	Polysomnography and actigraphy
Epigenetic regulators	MicroRNA	None identified

Aβ—amyloid beta-protein; APOE—apolipoprotein E; CSF—cerebrospinal fluid; FDG—fluorodeoxyglucose; GAP-43—growth-associated protein 43; GFAP—glial fibrillary acidic protein; ICAM-1—intercellular adhesion molecule-1; MRI—magnetic resonance imaging; HSD-1—hydroxysteroid dehydrogenase—1; MCP1—monocyte chemotactic protein-1;NAA—N-acetylaspartic acid; PET—positron emission tomography; RNA—ribonucleic acid; SNAP25—synaptosomal-associated protein, 25 kDa; SPECT—single-photon emission computed tomography; sTRM2—soluble triggering receptor expressed on myeloid cell 2; SV2A—synaptic vesicle glycoprotein 2A; TSPO—translocator protein; p-tau—phosphorylated tau; VCAM-1—vascular cell adhesion molecule-1; VAChT—vesicular acetylcholine transporters; VILIP-1—visinin-like protein-1.

**Table 3:** Amyloid, tau, neurodegeneration (AT(N)) biomarkers.

	Amyloid (A)	Tau (T)	Neurodegeneration (N)
Imaging	Amyloid PET	Tau PET	FDG PET; MRI; spectroscopy
CSF	Aβ 42/40	p-tau (181, 217)	Total tau; NfL; VILIP-1
Plasma	Aβ 42/40	p-tau (181, 217)	Total tau; NfL

Aβ—amyloid-beta protein; CSF—cerebrospinal fluid; FDG—fluorodeoxyglucose; MRI—magnetic resonance imaging; NfL—neurofilament light; PET—positron emission tomography; p-tau—phospho-tau.

**Table 4:** Pathways of biomarkers to progress to clinical use.

Pathway	Characteristic
Companion diagnostic	Required for appropriate use of a specific agent
In vitro diagnostic device (IVD)	Review by the FDA varies according to level of risk associated with the biomarker
510(k) pathway	Shown to be substantially equivalent to an approved IVD with performance characteristics at least as good as the approved IVD
Laboratory Developed Test (LDT)	Performed in a single laboratory; relatively limited FDA review
Research Use Only (RUO)	Cannot be used in diagnosis; may be used to gather additional information on the biomarker

## Data Availability

Not applicable.
